# Risk Factors for Acquisition of Drug Resistance during Multidrug-Resistant Tuberculosis Treatment, Arkhangelsk Oblast, Russia, 2005–2010

**DOI:** 10.3201/eid2106.141907

**Published:** 2015-06

**Authors:** Sarah E. Smith, Julia Ershova, Natalia Vlasova, Elena Nikishova, Irina Tarasova, Platon Eliseev, Andrey O. Maryandyshev, Igor G. Shemyakin, Ekaterina Kurbatova, J. Peter Cegielski

**Affiliations:** Centers for Disease Control and Prevention, Atlanta, Georgia, USA (S.E. Smith, J. Ershova, E. Kurbatova, J.P. Cegielski);; Arkhangelsk Regional Tuberculosis Dispensary, Arkhangelsk, Russia (N. Vlasova, E. Nikishova, I. Tarasova, P. Eliseev, A.O. Maryandyshev);; Northern State Medical University, Arkhangelsk (A.O. Maryandyshev);; State Research Center for Applied Microbiology and Biotechnology, Obolensk, Russia (I.G. Shemyakin)

**Keywords:** tuberculosis and other mycobacteria, antimicrobial resistance, Russia, acquired drug resistance, risk factors, bacteria

## Abstract

Acquired resistance to antituberculosis drugs decreases effective treatment options and the likelihood of treatment success. We identified risk factors for acquisition of drug resistance during treatment for multidrug-resistant tuberculosis (MDR TB) and evaluated the effect on treatment outcomes. Data were collected prospectively from adults from Arkhangelsk Oblast, Russia, who had pulmonary MDR TB during 2005–2008. Acquisition of resistance to capreomycin and of extensively drug-resistant TB were more likely among patients who received <3 effective drugs than among patients who received >3 effective drugs (9.4% vs. 0% and 8.6% vs. 0.8%, respectively). Poor outcomes were more likely among patients with acquired capreomycin resistance (100% vs. 25.9%), acquired ofloxacin resistance (83.6% vs. 22.7%), or acquired extensive drug resistance (100% vs. 24.4%). To prevent acquired drug resistance and poor outcomes, baseline susceptibility to first- and second-line drugs should be determined quickly, and treatment should be adjusted to contain >3 effective drugs.

## Abstract 2

Resistance should be determined quickly, and treatment should contain at least 3 effective drugs.

Treatment of multidrug-resistant tuberculosis (MDR TB) is complicated by the length of treatment, toxicity, and expense involved in use of second-line drugs. The latest World Health Organization (WHO) recommendations for treatment of MDR TB include use of a second-line injectable agent for 8 months, a fluoroquinolone, pyrazinamide, and >2 additional effective second-line drugs for almost 2 years (*1*). Fluoroquinolones and second-line injectable agents are essential for treatment of MDR TB because of their bactericidal activity relative to other second-line drugs (*2*,*3*). The second-line companion drugs are bacteriostatic and are used mainly to prevent amplification of resistance to the 2 key bactericidal drugs (*4*–*6*).

*Mycobacterium tuberculosis* drug resistance occurs by 2 mechanisms: initial infection with a resistant strain (primary resistance) or emergence of a resistant population of bacilli in a patient who initially had drug-susceptible TB (acquired resistance). Acquired drug resistance develops when inadequate treatment kills drug-susceptible *M. tuberculosis* bacilli while allowing bacilli with spontaneously occurring mutations that confer drug resistance to flourish until they predominate (*7*). Inadequate treatment can be a consequence of insufficient dosing, poor gastrointestinal absorption of oral medications, substandard quality of drugs, poor adherence to treatment, unsatisfactory duration of treatment, or treatment with a regimen containing >1 drugs to which the organism is already resistant (*7*,*8*). Acquisition of additional drug resistance, especially to fluoroquinolones or second-line injectable agents, leaves few treatment options, complicating an already difficult treatment (*9*).

WHO estimates that almost half of all TB cases in countries of the former Soviet Union involve resistance to >1 drug and that 1 in 5 TB patients has MDR TB (*10*). Furthermore, in this region, prevalence of extensively drug-resistant (XDR) TB, defined as MDR TB with additional resistance to any fluoroquinolone and >1 of the 3 second-line injectable agents, is among the highest in the world (*10*,*11*). In Russia, the proportion of new cases that are MDR TB varies from 8.8% to 15% across regions (*10*). Reported proportions of MDR TB in new (13.5%–19%) and previously treated (45%–60%) case-patients have been among the highest in Arkhangelsk Oblast, which is in northwestern Russia (*12*,*13*). Although the overall rate of TB notification in this oblast is declining, especially among new cases, the relative proportion of MDR TB is increasing (*14*).

Before 2000, the Arkhangelsk TB Control Program had limited access to second-line drugs because of their high cost (*15*). In early 2002, this program applied to the Green Light Committee (GLC), which was created to evaluate, lend guidance, and approve TB control programs for access to reduced-price, quality-assured, second-line drugs (*16*). The GLC led the development of WHO guidelines for programmatic management of drug-resistant TB, at the time called DOTS–Plus. The GLC required programs to follow these guidelines, which were designed to minimize the risk for acquired drug resistance for MDR TB patients and to improve treatment outcomes (*17*). In May 2003, the GLC approved Arkhangelsk TB Control Program procurement of quality-assured second-line drugs.

Even programs that follow WHO guidelines for programmatic management of drug-resistant TB have reported detection of XDR TB in patients undergoing treatment for MDR TB, suggesting that *M. tuberculosis* is acquiring additional drug resistance over the course of treatment (*18*). Our goals with this study were to determine the frequency of acquired drug resistance, the risk factors for acquisition of additional drug resistance over the course of MDR TB treatment, and which treatment regimens for MDR TB will decrease the risk for acquired resistance and lead to better treatment outcomes.

## Methods

### Study Population and Data Collection

The study prospectively enrolled 2 cohorts of consecutively seen, consenting, nonimprisoned adult patients in Arkhangelsk Oblast, Russia, who had confirmed pulmonary MDR TB and were starting treatment with second-line drugs. MDR TB was confirmed by sputum culture and drug-susceptibility testing (DST) at the regional TB laboratory. Patients in cohort 1 were enrolled from January 1, 2005, through December 31, 2006; patients in cohort 2 were enrolled from January 1, 2007, through December 31, 2008. The 2 cohorts were approved in 2 separate applications to the GLC. Most patients in cohort 1 had been on a waiting list for MDR TB treatment for an extended amount of time at the time of treatment initiation, whereas most patients in cohort 2 had a recent diagnosis of MDR TB at the time of treatment initiation. Study inclusion criteria required having >1 *M. tuberculosis*–positive culture result within 1 month (before or after) of starting second-line drugs for the treatment for MDR TB (baseline isolate) and >1 month of treatment with second-line drugs.

Standardized forms were used to prospectively collect sociodemographic, clinical, and laboratory data from patients’ medical charts. Chest radiographs were read by experienced chest physicians and radiologists, and results were recorded in a standardized manner. Sputum specimens were collected from each patient at the start of second-line drug treatment (baseline isolate) and then monthly until treatment outcome was known.

The study protocol was approved by ethics committees at the US Centers for Disease Control and Prevention (CDC), Northern State Medical University in Arkhangelsk, and the State Research Center for Applied Microbiology and Biotechnology (SRCAMB) in Obolensk, Russia. All patients provided written informed consent.

### Laboratory Methods

Baseline and follow-up sputum specimens were cultured on Lowenstein-Jensen solid media in the Arkhangelsk Regional TB Dispensary Laboratory. Frozen *M. tuberculosis* isolates were shipped to SRCAMB in Obolensk, Russia, for first- and second-line DST, genotyping, and DNA sequencing. The analysis reported in this article is based on the SRCAMB results. Testing at SRCAMB was conducted months to years after patients were enrolled, and results were not available in real time.

At SRCAMB, each isolate was cultured in 6 mL of Middlebrook 7H9 broth to an optical density of >1.0 McFarland standard and on Lowenstein-Jensen medium. Susceptibility testing for the baseline and the last follow-up (final) isolates from each patient were determined for isoniazid, rifampin, ethambutol, streptomycin, kanamycin, amikacin, capreomycin, ofloxacin, ethionamide, and para-aminosalicylic acid. Drug susceptibility was determined by the proportion method according to CDC protocol (*19*). When drug susceptibility of a patient’s baseline and final isolates differed, isolates were genotyped by mycobacterial interspersed repetitive units–variable number of tandem repeats analysis and by restriction fragment-length polymorphism–IS6110 analysis to determine whether the isolates were the same strain.

### Definitions

Definitions of pulmonary TB, MDR TB, and treatment outcomes were based on WHO guidelines (*20*). Third-line drugs refer to drugs classified by WHO as group 5 drugs (*1*). “Effective treatment” was defined as treatment with a drug or combination of drugs to which baseline DST reported susceptibility. “Ineffective treatment” was considered use of said drug(s) despite reported resistance. Effectiveness of treatment was considered unknown and the patient was not included in the analysis when the patient never received said drugs or baseline DST results were not available. “Acquired resistance” was defined as occurring when baseline DST result showed susceptibility in vitro, the final DST result showed resistance in vitro, and genotypes matched for the initial and final isolates. Acquired resistance was considered absent in each of the following 3 scenarios: 1) baseline and final isolates were susceptible to a drug; 2) DST result changed from susceptible to resistant, but genotyping indicated different strains; or 3) no follow-up positive culture results were available because the patient’s sputum culture results sustainably converted to negative after the baseline DST, the patient died, or the patient was lost to follow-up. In each instance, the denominator for each group refers to the number of isolates with baseline DST results indicating susceptibility to the given drug. Patients whose baseline isolate was resistant to a given drug were excluded from the acquired resistance analysis for that drug. Successful treatment outcome was defined as cure and treatment completion (*20*). Poor treatment outcome was defined as treatment failure or death. The second-line companion drugs were para-aminosalicylic acid or ethionamide. Being underweight was defined as having a body mass index <18.5 kg/m^2^.

### Data Management and Statistical Analyses

The data from standardized forms were double-entered into an Epi Info (CDC, Atlanta, GA, USA) database in Arkhangelsk and sent to CDC for checking and analysis. Laboratory data were sent directly from the SRCAMB laboratory to CDC.

Statistical analyses were performed by using SAS version 9.3 (SAS Institute Inc., Cary, NC, USA). The main outcome of interest was acquired resistance to specific second-line drugs. The secondary outcome of interest was end-of-treatment outcome. Bivariate associations between the potential risk factors and the outcome variable for each respective analysis were examined by using the Fisher exact test with a significance level of 0.05. Multivariate logistic regression was used to assess associations of acquired resistance and treatment with treatment outcomes while controlling for the potential confounding effects of extent of drug resistance at baseline, disease severity, and previous treatment for MDR TB.

## Results

### Patient Population

A total of 202 MDR TB patients were enrolled in the study: 81 in cohort 1 and 121 in cohort 2. Median patient age was 42 years, 171 (84.7%) patients were male, and none were HIV infected (HIV test results were available for all patients). Most patients had previously received treatment for TB: 69 (34.7%) had received first-line drugs and 73 (36.7%) had received additional second-line drugs. Almost all patients (189 [93.6%]) had pulmonary cavities, 162 (80.2%) had bilateral lung involvement, 161 (80.9%) had sputum smears with acid-fast bacilli seen with microscopy, and 45 (22.3%) had a body mass index <18.5 kg/m^2^ at the start of MDR TB treatment.

A total of 740 *M. tuberculosis* isolates from 202 patients were shipped to SRCAMB. Of these 202 patients, baseline DST results from SRCAMB were available for 171 (84.7%) and were included in analysis of baseline drug resistance (Figure). Of the 171 patients for whom baseline DST results were available, follow-up DST results at SRCAMB were available for 117. Among the other 54 patients (without a final DST result), cultures converted after the initial isolate for 45, follow-up cultures were contaminated or did not grow for 5, and a reason was not documented for 4.

**Figure:**
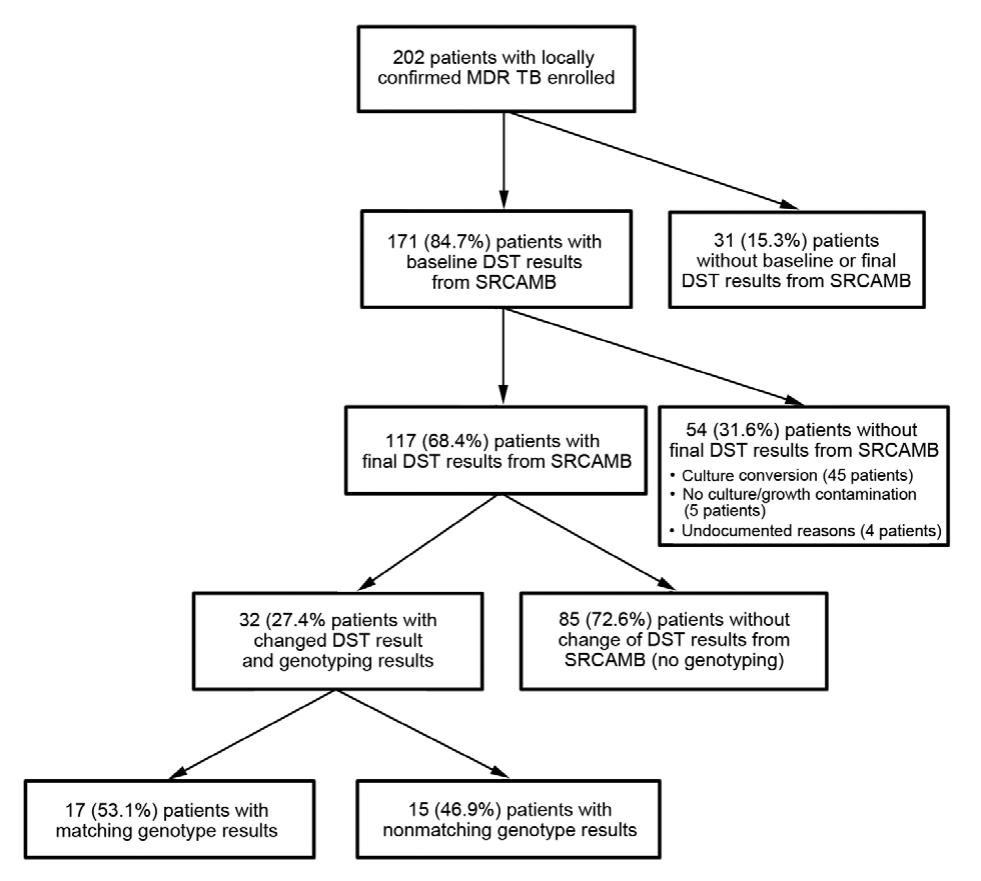
Patient enrollment and reference laboratory drug susceptibility and genotype testing results. DST, drug-susceptibility testing; MDR TB, multidrug-resistant tuberculosis; SRCAMB, State Research Center for Applied Microbiology and Biotechnology.

### Baseline Drug Resistance

Among 171 patients for whom baseline isolate DST results were available (Table 1), MDR TB was not confirmed by DST for 4 (2.3%) isolates, and 130 (76.0%) baseline isolates were resistant to 4 first-line drugs tested (rifampin, isoniazid, ethambutol, and streptomycin). In addition, 74 isolates were resistant to >1 of the 3 second-line injectable agents: 72 (42.1%) to kanamycin, 30 (17.5%) to amikacin, 13 (7.6%) to capreomycin, and 10 (5.8%) to all 3. A total of 10 (5.8%) isolates were resistant to ofloxacin, and 7 (4.1%) were XDR. Of the second-line companion drugs tested, resistance to ethionamide was found for 46 (26.9%) isolates and to para-aminosalicylic acid for 54 (31.6%) isolates.

**Table 1:** Drug susceptibility and acquired resistance of *Mycobacterium tuberculosis* from 171 patients with MDR TB, Arkhangelsk Oblast, Russia, 2005–2010*

Drug(s) tested	Baseline, no. (%)		Acquired resistance no. (% of susceptible)
	Resistant	Susceptible	
RIF	167 (97.7)	4 (2.3)	1 (25.0)
INH	170 (99.4)	1 (0.6)	0
MDR TB drugs†	167 (97.7)	4 (2.3)	1 (25.0)
EMB	135 (79.0)	36 (21.0)	2 (5.6)
STR	163 (95.3)	8 (4.7)	1 (12.5)
All 4 first-line drugs‡	130 (76.0)	41 (24.0)	3 (7.3)
KAN	72 (42.1)	99 (57.9)	4 (4.0)
AMK	30 (17.5)	141 (82.5)	1 (0.7)
CAP	13 (7.6)	158 (92.4)	3 (1.9)
Any second-line injectable§	74 (43.3)	97 (56.7)	Not applicable
All 3 second-line injectables	10 (5.8)	161 (94.2)	7 (4.4)
OFX	10 (5.8)	161 (94.2)	6 (3.7)
XDR TB drugs¶	7 (4.1)	164 (95.9)	4 (2.4)
ETA	46 (26.9)	125 (73.1)	6 (4.8)
PAS	54 (31.6)	117 (68.4)	2 (1.7)
Both second-line companion drugs	18 (10.5)	153 (89.5)	6 (3.9)
All second-line drugs	1 (0.6)	170 (99.4)	14 (8.2)
All drugs	1 (0.6)	170 (99.4)	16 (9.4)

*AMK, amikacin; CAP, capreomycin; EMB, ethambutol; ETA, ethionamide; FQ, fluoroquinolone; INH, isoniazid; KAN, kanamycin; OFX, ofloxacin; MDR TB, multidrug-resistant tuberculosis; RIF, rifampin; STR, streptomycin; PAS, para-aminosalicylic acid; XDR, extensively drug-resistant tuberculosis.  †RIF and INH. ‡RIF, INH, EMB, and STR. §KAN,AMK, and CAP. ¶RIF, INH, a second-line injectable drug, and an FQ.

### Acquired Drug Resistance

Among 117 patients for whom final DST results from SRCAMB were available, results for the baseline and final isolates differed for 32 (27.3%) and the isolates were successfully genotyped. Of these, genotype results for isolate pairs matched for 17 (53.1%) patients and were eligible for inclusion in numerators for respective analyses of acquired drug resistance (Figure). Of 41 paired isolates with baseline susceptibility to >1 first-line drug, resistance to a first-line drug was acquired in 3 (7.3%) (Table 1). Of 161 paired isolates with baseline susceptibility to >1 second-line injectable agents, resistance to >1 second-line injectable agents was acquired in 7 (4.4%): resistance to kanamycin by 4 (4.0%) of 99, to amikacin by 1 (0.7%) of 141, and to capreomycin by 3 (1.9%) of 158. Resistance to ofloxacin was acquired by 6 (3.7%) of 161, and XDR TB was acquired by 4 (2.4%) of 164 over the course of treatment for MDR TB.

### Risk Factors for Acquisition of Drug Resistance

Acquired resistance to capreomycin was significantly associated with receiving the following drugs or drug groups: <3 effective drugs (p = 0.008), an ineffective fluoroquinolone (p = 0.009), or ineffective para-aminosalicylic acid (p = 0.02). Furthermore, acquired resistance to capreomycin was associated with not having received ofloxacin (regardless of baseline DST results) (p = 0.003), baseline resistance to ofloxacin (p = 0.008), and baseline resistance to para-aminosalicylic acid (p = 0.03) (Table 2). In addition, patients whose isolates acquired resistance to capreomycin were more likely to have received moxifloxacin (instead of ofloxacin) in the treatment regimen.

**Table 2:** Risk factors for acquired resistance to CAP while receiving treatment for MDR TB, 158 patients, Arkhangelsk Oblast, Russia, 2005–2010*

Variable†	Total	Acquired capreomycin resistance, no. (%)		p value‡
		Yes	No	
Received ≥3 effective drugs				
Yes	126	0	126 (100)	0.008
No	32	3 (9.4)	29 (90.6)	
Ever received effective FQ treatment§				
Yes	148	1 (0.7)	147 (99.3)	0.009
No	9	2 (22.2)	7 (77.8)	
Ever received effective PAS treatment§				
Yes	110	0 (0)	110 (100)	0.02
No	46	3 (6.5)	43 (93.5)	
Previous treatment with FQ				
Yes	28	2 (7.1)	26 (92.9)	0.08
No	130	1 (0.8)	129 (99.2)	
Previous PAS treatment¶				
Yes	23	2 (8.7)	21 (91.3)	0.08
No	112	1 (0.9)	111 (99.1)	
First time patient treated for MDR TB				
Yes	134	1 (0.7)	133 (99.3)	0.06
No	24	2 (8.3)	22 (91.7)	
Baseline ofloxacin DST result				
Resistant	9	2 (22.2)	7 (77.8)	0.008
Susceptible	149	1 (0.7)	148 (99.3)	
Baseline PAS DST result				
Resistant	47	3 (6.4)	44 (93.6)	0.03
Susceptible	111	0 (0)	111 (100)	
Received OFX during episode				
Yes	135	0 (0)	135 (100)	0.003
No	23	3 (13)	20 (87)	
Received MOX during episode				
Yes	31	3 (9.7)	28 (90.3)	0.007
No	127	0 (0)	127 (100)	

*CAP, capreomycin; DST, drug-susceptibility test; FQ, fluoroquinolone; MDR TB, multidrug-resistant tuberculosis; MOX, moxifloxacin; OFX, ofloxacin; PAS, para-aminosalicylic acid.  †Certain variables were tested for association but omitted from table because results were not statistically significant at α = 0.1. ‡Fisher exact test. §Patient(s) who did not receive treatment with the respective drug during the current episode of MDR TB were not included in the analysis. ¶For 23 patients, history of treatment with PAS was unknown.

Acquired resistance to ofloxacin was significantly more common among patients who were underweight (p = 0.02) (Table 3). Patients with acquired ofloxacin resistance were more likely to have received moxifloxacin (p = 0.006), to have had fluoroquinolones switched during treatment (p = 0.05), and to be receiving a third-line drug during the current episode (p = 0.01).

**Table 3:** Risk factors for acquiring resistance to OFX during MDR TB treatment, 161 patients, in Arkhangelsk, Russia, 2005–2010*

Variable†	Total	Acquired ofloxacin resistance, no. (%)		p value‡
		Yes	No	
Enrollment cohort				
2005–2006	64	0 (0)	64 (100)	0.08
2007–2008	97	6 (6.2)	91 (93.8)	
Body mass index <18.5 at MDR TB diagnosis				
Yes	35	4 (11.4)	31 (88.6)	0.02
No	126	2 (1.6)	124 (98.4)	
Hospitalized at time of enrollment				
Yes	159	5 (3.1)	154 (96.9)	0.07
No	2	1 (50)	1 (50)	
Ever received MOX during current episode				
Yes	26	4 (15.4)	22 (84.6)	0.007
No	135	2 (1.5)	133 (98.5)	
Changed FQ during current episode				
Yes	10	2 (20)	8 (80)	0.05
No	151	4 (2.6)	147 (97.4)	
Ever received a third-line drug during episode				
Yes	79	6 (7.6)	73 (92.4)	0.01
No	82	0 (0)	82 (100)	

*FQ, fluoroquinolone; OFX, ofloxacin; MDR TB, multidrug-resistant tuberculosis; MOX, moxifloxicin.  †Certain variables were tested for association but omitted from table because results were not statistically significant at α = 0.1. ‡Fisher exact test.

Acquired XDR TB was more frequent among those receiving <3 effective drugs than among those receiving >3 effective drugs (p = 0.03) and among those who were underweight (p = 0.03) (Table 4). Those who acquired XDR TB were more likely to be receiving moxifloxacin during the current episode (p = 0.02). Patients in whom isolates acquired resistance to any second-line companion drug (ethionamide or para-aminosalicylic acid) were less likely to have received ofloxacin (p = 0.003) and more likely to be receiving moxifloxacin (p<0.001) during the current episode (Table 5).

**Table 4:** Risk factors for acquiring extensive drug resistance during MDR TB treatment, 164 patients, Arkhangelsk, Russia, 2005–2010*

Variable†	Total	Acquired extensive drug resistance, no. (%)		p value‡
		Yes	No	
Treated with ≥3 effective drugs				
Yes	129	1 (0.8)	128 (99.2)	0.03
No	35	3 (8.6)	32 (91.4)	
Ever received effective FQ§				
Yes	160	3 (1.9)	157 (98.1)	0.07
No	3	1 (33.3)	2 (66.7)	
Body mass index <18.5 at MDR TB diagnosis				
Yes	36	3 (8.3)	33 (91.7)	0.03
No	128	1 (0.8)	127 (99.2)	
Baseline OFX susceptibility result				
Resistant	3	1 (33.3)	2 (66.7)	0.07
Susceptible	161	3 (1.9)	158 (98.1)	
Ever received OFX during current episode				
Yes	144	2 (1.4)	142 (98.6)	0.07
No	20	2 (10)	18 (90)	
Ever received MOX during current episode				
Yes	29	3 (10.3)	26 (89.7)	0.02
No	135	1 (0.7)	134 (99.3)	

*FQ, fluoroquinolone; OFX, ofloxacin; MDR TB, multidrug-resistant tuberculosis; MOX, moxifloxicin  †Certain variables were tested for association but omitted from table because results were not statistically significant at α = 0.1. ‡Fisher exact test. §Patients who did not receive treatment with the respective drug during the current episode of MDR TB were not included in the analysis.

**Table 5:** Risk factors for acquiring resistance to second-line companion drugs during MDR TB treatment, 153 patients, Arkhangelsk Oblast, Russia, 2005–2010*

Variable†	Total	Acquired resistance to ETA or PAS, no. (%)		p value‡
		Yes	No	
Enrollment cohort				
2005–2006	58	0 (0)	58 (100)	0.08
2007–2008	95	6 (6.3)	89 (93.7)	
Thoracic surgery during current episode				
Yes	2	1 (50)	1 (50)	0.08
No	151	5 (3.3)	146 (96.7)	
Ever received OFX during current episode				
Yes	133	2 (1.5)	131 (98.5)	0.003
No	20	4 (20)	16 (80)	
Ever received MOX during current episode				
Yes	28	5 (17.9)	23 (82.1)	<0.001
No	125	1 (0.8)	124 (99.2)	
Ever received a third-line drug during current episode				
Yes	77	6 (7.8)	71 (92.2)	0.03
No	76	0 (0)	76 (100)	

*OFX, ofloxacin; MDR TB, multidrug-resistant tuberculosis; MOX, moxifloxacin. †Certain variables were tested for association but omitted from table because results were not statistically significant at α = 0.1. ‡Fisher exact test.

### Treatment Outcomes

Of 171 patients for whom baseline DST results were available, treatment was successfully completed for 94 (55.0%), treatment failed for 18 (10.5%), 20 (11.7%) died, and 39 (22.8%) defaulted from treatment. Poor treatment outcomes (treatment failure or death) were more likely among patients whose MDR TB acquired resistance to capreomycin (100% vs. 25.9%; p = 0.02) or ofloxacin (83.3% vs. 22.7%; p = 0.004) or became XDR TB (100% vs. 24.4%; p = 0.004) than among those in whom the respective resistance was not acquired (Table 6). Patients who received an effective fluoroquinolone were statistically less likely to have poor treatment outcomes than were those who received an ineffective fluoroquinolone (25.6% vs. 85.7%; p = 0.002). Patients who received any third-line drug were more likely to have previously received treatment for MDR TB (21.6% vs. 8.4%; p = 0.02), have resistance to >4 drugs at baseline (72.7% vs. 47.0%; p<0.001), and experience treatment failure or die (42.0% vs. 20.6%; p = 0.01) than those who did not receive any third-line drug. According to multivariable analysis, compared with no acquired resistance, acquired resistance to ofloxacin was associated with 10.2-fold (95% CI 1.1–95.1) increased odds of poor outcome when confounding was controlled for (Table 6). Compared with not receiving a third-line drug, treatment with a third-line drug was associated with 2.7-fold (95% CI 1.2–5.7) increased odds of poor treatment outcome when confounding was controlled for. Compared with not receiving effective fluoroquinolone treatment, effective treatment with a fluoroquinolone was associated with 16.7-fold (95% CI 1.9–100.0) increased the odds of successful treatment outcome when confounding was controlled for.

**Table 6:** Effect of acquired resistance and treatment on MDR TB treatment outcomes, 132 patients, Arkhangelsk Oblast, Russia, 2005–2010*

Variable†	Total	Successful treatment outcome, no. (%)‡	Poor treatment outcome, no. (%)§	Fisher exact p value	aOR (95% CI)¶
Overall	132	94 (71.2)	38 (28.8)		
Acquired resistance to any second-line drug#					
Yes	13	6 (46.2)	7 (53.8)	0.05	1.93 (0.54–6.88)
No	118	88 (74.6)	30 (25.4)		
Acquired resistance to CAP#					
Yes	3	0	3 (100)	0.02	NR
No	116	86 (74.1)	30 (25.9)		
Acquired resistance to OFX#					
Yes	6	1 (16.7)	5 (83.3)	0.004	10.18 (1.09–95.08)
No	119	92 (77.3)	27 (22.7)		
Acquired XDR#					
Yes	4	0 (0)	4 (100)	0.004	NR
No	123	93 (75.6)	30 (24.4)		
Ever received effective FQ**					
Yes	125	93 (74.4)	32 (25.6)	0.002	0.06 (0.01–0.53)††
No	7	1 (14.3)	6 (85.7)		
Ever received CAP during current episode					
Yes	104	67 (64.4)	37 (35.6)	0.08	1.42 (0.49–4.05)
No	33	27 (81.8)	6 (18.2)		
Ever received MOX during current episode					
Yes	27	14 (51.9)	13 (48.1)	0.06	1.48 (0.56–3.90)
No	110	80 (72.7)	30 (27.3)		
Ever received third-line drug during current episode					
Yes	69	40 (58)	29 (42)	0.01	2.68 (1.25–5.75)
No	68	54 (79.4)	14 (20.6)		

*aOR, adjusted odds ratio; CAP, capreomycin; FQ, fluoroquinolone; MDR TB, multidrug-resistant tuberculosis; MOX, moxifloxacin; NR, no result; OFX, ofloxacin; XDR, extensively drug-resistant tuberculosis.  †Certain variables were tested for association but omitted from table because results were not statistically significant at α = 0.1. ‡Cure or treatment completion. §Death or treatment failure. ¶aOR for poor treatment outcome versus successful treatment outcome controlling for extent of drug resistance at baseline, severity of disease, and previous treatment for MDR TB. #Patient(s) with baseline TB resistance to the respective drug (or drug groups) were not included in the analysis. **Patient(s) who did not receive treatment with the respective drug during the current episode of MDR TB were not included in the analysis ††16.67 (95% CI 1.89–100.0) aOR of successful treatment outcome vs. poor treatment outcome.

## Discussion

This study measured the frequency with which drug resistance was acquired during MDR TB treatment and identified statistically significant associations for acquiring resistance to a specific drug or group of drugs in a population of MDR TB patients being managed in a high-quality TB program. The rates of acquired resistance to the 2 essential groups of drugs for MDR TB treatment were 4.3% for second-line injectable agents and 3.7% for fluoroquinolones, the middle of the range recently reported for GLC-approved programs (*21*). In this study, odds of treatment failure or death were 10.2-fold higher among those with acquired resistance to ofloxacin than among those without, further supporting the value of this class of drugs in successful MDR TB treatment. Although this study focused on 1 region of Russia, it reflects the broader global context of increasing use of second-line drugs and rapidly emerging resistance as exemplified by the global phenomenon of XDR TB reported in 2006 (*22*,*23*).

We found that the highest proportion of acquired second-line drug resistance was to any second-line injectable agent (4.3%), most frequently kanamycin (4.0%). Given the high baseline level of kanamycin resistance, the cross-resistance between second-line injectable agents, and the rate of acquired resistance to second-line injectable agents illustrated in this study, treating MDR TB with second-line injectable agents is becoming less of an effective option (*24*). Furthermore, because of the common baseline resistance to kanamycin, most of the acquired XDR TB was the result of acquired ofloxacin resistance. Historically in Arkhangelsk Oblast, kanamycin was widely used for TB treatment along with 2–3 other drugs, including first- and second-line drugs, whereas fluoroquinolones were rarely used for TB treatment before GLC approval in 2003 (*12*,*25*). Acquired resistance to fluoroquinolones during MDR TB treatment was reported for 11.2% of cases in 9 countries, including Russia, possibly because of the high mutation frequency of the *gyrA* and *gyrB* genes (*21*,*26*–*28*). Of any single second-line drug tested in this study, acquired resistance to ofloxacin occurred second most often. Most patients whose isolates acquired resistance to either of the second-line companion drugs tested also experienced acquired resistance to a second-line injectable agent, ofloxacin, or both (i.e., acquired extensive drug resistance), making TB in these patients virtually untreatable with available drugs (*28*).

As seen elsewhere and in this population of MDR TB patients for whom prevalence of baseline resistance to kanamycin, ethionamide, and para-aminosalicylic acid was high, baseline susceptibility to and use of fluoroquinolones were essential for preventing further resistance to second-line injectable agents, preventing acquired XDR TB, and increasing treatment success (*29*,*30*). With fewer effective treatment options, the risk for acquired resistance to additional drugs increases (*28*). This study illustrates the value of effective use of bactericidal drugs such as fluoroquinolones and companion drugs (especially para-aminosalicylic acid) in preventing acquired resistance to second-line injectable agents during treatment for MDR TB. Other studies reported a significant association between use of thioamides and treatment success but not with para-aminosalicylic acid (*31*).

WHO recommends that MDR TB be treated with >4 second-line drugs to which *M. tuberculosis* is likely to be susceptible plus pyrazinamide, creating a regimen of >5 drugs during the intensive phase of treatment (*1*). Many factors make creating such a regimen challenging, including availability of timely DST results for second-line drugs and availability of multiple drugs within a class of second-line drugs. In this setting, in which baseline DST results for multiple second-line drugs were available, *M. tuberculosis* treated with >3 effective drugs were less likely to acquire resistance to each of the drugs or drug groups tested than were *M. tuberculosis* treated with <3 effective drugs. However, this association was only statistically significant for acquired resistance to capreomycin and for acquired extensive drug resistance. Treatment with >4 effective drugs had a similar, but not statistically significant, inverse association with acquired drug resistance.

Acquired resistance to ofloxacin was not associated with any of the effective treatment variables. The treatment and patient management characteristics that were associated with acquired ofloxacin resistance may be an artifact of clinical management practices when treatment regimens fail and probably reflect confounding. The treatment for severe disease or a failing regimen will often be switched to a newer generation fluoroquinolone because these are thought to be more effective and because cross-resistance within the class is not complete (*32*,*33*). Therefore, the only significant risk factor for acquired ofloxacin resistance in this population was being underweight, which is a risk factor for incident TB and an indicator of disease severity, regardless of drug susceptibility (*34*).

Other studies have found that the main risk factors for acquired drug resistance included empiric re-treatment (i.e., without reference to DST) and unsupervised treatment (*35*,*36*). In this study population, drug resistance was acquired among patients with MDR TB even though the patients had received individualized treatment, and directly observed therapy was mandatory for all patients in the program.

The MDR TB treatment outcomes for this population are consistent with previously reported outcomes. Treatment success for this population (55%) was greater than the WHO-reported worldwide average (48%) but less than published results of individualized MDR TB treatment programs (*31*,*37*). This study indicates that treatment failure and death are significantly more common among patients who experienced acquired resistance to capreomycin or ofloxacin or who acquired XDR TB than among patients who did not, providing even more evidence that these drugs are essential for successful treatment of MDR TB (*38*).

This study had several limitations. First, the relatively small sample size limited statistical power of our analyses. Second, testing of *M. tuberculosis* for susceptibility to second-line drugs is difficult and not well standardized (*39*), which could have caused patient misclassification for both the effective treatment and acquired resistance variables because both sets of variables involve DST results. Third, the effective treatment variables did not consider dosage or length of time the drug was given—all key components of effective treatment. Last, the prevalence of cavitary disease in this population was unusually high, and because cavitation is associated with acquired resistance, the results might not be directly applicable to patient populations with less chronic or destructive disease (*21*).

Knowing the drug resistance pattern in the community and risk factors for acquired resistance to second-line drugs can help TB programs initiate effective treatment regimens, prevent additional acquired resistance, and improve treatment outcomes for patients for whom MDR TB is suspected before DST results are available. The likelihood of treatment success can be further improved by adjusting treatment after receipt of DST results for second-line drugs. The need for rapid diagnosis of drug resistance and effective treatment is crucial.
